# Distinct magneto-Raman signatures of spin-flip phase transitions in CrI_3_

**DOI:** 10.1038/s41467-020-17320-3

**Published:** 2020-08-03

**Authors:** Amber McCreary, Thuc T. Mai, Franz G. Utermohlen, Jeffrey R. Simpson, Kevin F. Garrity, Xiaozhou Feng, Dmitry Shcherbakov, Yanglin Zhu, Jin Hu, Daniel Weber, Kenji Watanabe, Takashi Taniguchi, Joshua E. Goldberger, Zhiqiang Mao, Chun Ning Lau, Yuanming Lu, Nandini Trivedi, Rolando Valdés Aguilar, Angela R. Hight Walker

**Affiliations:** 1 Nanoscale Device Characterization Division, Physical Measurement Laboratory, National Institute of Standards and Technology, Gaithersburg, MD 20899 USA; 2 Department of Physics, The Ohio State University, Columbus, OH 43210 USA; 3 Department of Physics, Astronomy, and Geosciences, Towson University, Towson, MD 21252 USA; 4 Materials Measurement Science Division, Material Measurement Laboratory, National Institute of Standards and Technology, Gaithersburg, MD 20899 USA; 5 Department of Physics, The Pennsylvania State University, University Park, PA 16802 USA; 6 Department of Physics, University of Arkansas, Fayetteville, AR 72701 USA; 7 Department of Chemistry and Biochemistry, The Ohio State University, Columbus, OH 43210 USA; 8 National Institute for Materials Science, 1-1 Namiki, Tsukuba, Ibaraki 305-0044 Japan; 9 Present Address: Battery and Electrochemistry Laboratory, Institute of Nanotechnology, Karlsruhe Institute of Technology, 76344 Eggenstein-Leopoldshafen, Germany

**Keywords:** Magnetic properties and materials, Two-dimensional materials, Ferromagnetism, Magnetic properties and materials, Magneto-optics

## Abstract

The discovery of 2-dimensional (2D) materials, such as CrI_3_, that retain magnetic ordering at monolayer thickness has resulted in a surge of both pure and applied research in 2D magnetism. Here, we report a magneto-Raman spectroscopy study on multilayered CrI_3_, focusing on two additional features in the spectra that appear below the magnetic ordering temperature and were previously assigned to high frequency magnons. Instead, we conclude these modes are actually zone-folded phonons. We observe a striking evolution of the Raman spectra with increasing magnetic field applied perpendicular to the atomic layers in which clear, sudden changes in intensities of the modes are attributed to the interlayer ordering changing from antiferromagnetic to ferromagnetic at a critical magnetic field. Our work highlights the sensitivity of the Raman modes to weak interlayer spin ordering in CrI_3_.

## Abstract (web summary)

Thin samples CrI_3_ exhibit a phase transition under an applied magnetic field from layered antiferromagnetism to ferromagnetism. Here the authors observe an associated abrupt change in the magneto-Raman spectra, illustrating the sensitivity of Raman spectra to magnetic ordering.

## Introduction

Magnetic van der Waals-bonded materials represent a rapidly growing research field^1–6^, where these materials provide a solid-state platform to study a variety of exciting physics and potential applications of magnetism in two dimensions, including proximity effects, control using strain and gating, spin fluctuations, magnetic excitations, spintronics, and possible quantum spin liquids^7^. One material of particular interest is chromium tri-iodide (CrI_3_), a ferromagnet (FM) at bulk thicknesses below the Curie temperature (*T*_c_) but with the remarkable property of layered antiferromagnetism (AFM) in thin multilayers^2^. Although each individual layer is FM, the layers themselves are AFM coupled, and this effect persists for samples tens of layers thick. Furthermore, the interlayer spin arrangement in CrI_3_ can be switched between AFM and FM by an electric field^8–10^, applied pressure^11^, and a magnetic field^2,12–15^, providing tunability in potential devices.

Raman spectroscopy is a powerful technique to study a variety of phenomena in 2D quantum materials, including effects of strain^16^, electron–phonon coupling^17^, phase transitions^18^, spin-phonon coupling^19^, and magnetic excitations^20–22^. In addition, the diffraction-limited spot size allows for the investigation of atomically thin samples and heterostructures using a non-contact probe. In this work, we collect temperature- and magnetic field (*B*)-dependent Raman spectra on a thin (~10 layers) CrI_3_ single crystal encapsulated in hexagonal boron nitride (hBN). Interestingly, at low temperature, increasing the magnetic field results in dramatic changes in the Raman spectra, indicating a magnetic field-induced phase transition when the interlayer spin arrangement changes from AFM to FM. By calculating the phonon dispersion of CrI_3_ in both the FM and AFM state, we conclude the two modes that only appear in the AFM state are zone-folded phonons. This work validates magneto-Raman spectroscopy as a sensitive technique to probe interlayer magnetic ordering in quantum materials.

## Results

### Polarization-dependent Raman at zero magnetic field

**Fig. 1:**
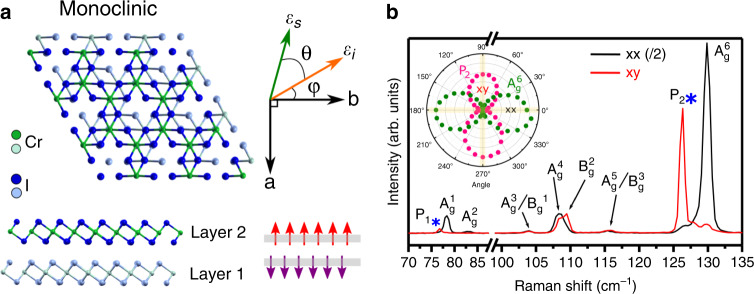
Crystal structure and Raman spectra of CrI_3_. **a** Top and side views of two layers of CrI_3_ with monoclinic structure and a schematic defining angles *θ* and *φ* with respect to the *a*- and *b*-crystal axes and the polarization vectors of the incoming *ε*_*i*_ and scattered *ε*_*s*_ light. The two CrI_3_ layers are color-coded differently. **b** Raman spectra for both xx (black) and xy (red) polarization configurations at *T* = 5 K and *B* = 0 T. Spectra in xx were divided by two for clarity. Peaks that appear below *T*_c_ are highlighted with blue asterisks. Inset shows intensity as a function of *θ* for P_2_ and ${\mathrm{A}}_{\mathrm{g}}^6$ in a polar plot.

A thin flake of CrI_3_ (≈7 nm from atomic force microscopy, or ≈10 layers) was encapsulated between two 20 nm and 30 nm flakes of hBN using the dry transfer technique^22,23^. In the ab plane, the Cr^3+^ atoms are arranged in a honeycomb lattice, where each chromium atom is bonded with six iodine atoms to form a distorted octahedron (see Fig. 1a). At room temperature, bulk CrI_3_ has a monoclinic structure (C2/m) but exhibits a crystallographic phase transition below ≈220 K to rhombohedral (R$\bar 3$)^24^, where the main difference between the two structures is the stacking of the layers (see Supplementary Fig. 1). The bulk *T*_c_ is ≈61 K^24^, with the spins aligned perpendicular to the ab plane. Surprisingly, the magnetic behavior of thinner samples is very different; whereas the spins still align perpendicular to the ab plane, the interlayer magnetic stacking is AFM, as demonstrated through a variety of experimental techniques^2,8,9,12–15,25,26^. It has been theoretically^27–30^ and experimentally^26,30^ suggested that atomically thin CrI_3_ does not go through the crystallographic phase transition that the bulk does, but instead remains in the monoclinic structure at low temperatures, resulting in AFM interlayer stacking.

Raman spectra were collected with a triple grating spectrometer using an excitation laser wavelength of 632.8 nm and keeping the power below 150 μW (≈1 µm spot size) at the sample to avoid heating. The laser polarization of the incoming *ε*_*i*_ light makes an angle *φ* with respect to the *b* axis (Fig. 1a), and the scattered *ε*_*s*_ light angle *θ* is changed from *θ* = 0° (parallel, xx) to *θ* = 90° (perpendicular, xy*)*. As the crystallographic *a*- and *b* axes are not known in our sample, the angle *φ* is an arbitrary, yet constant angle in our experiments. Figure 1b shows the Raman spectra at *T* = 5 K, in both the xx and xy polarization configurations. We confirm the monoclinic symmetry in our ≈10 layer flake by resolving two peaks at 108 cm^−1^ and 109 cm^−1^ between xx (black, scaled by 0.5) and xy (red), unlike the doubly degenerate peak seen in the rhombohedral structure^31^. Thus, we label the phonons using the irreducible representations of the 2/m point group, where only the A_g_ and B_g_ modes are Raman active^32,33^. The density functional theory (DFT) calculated atomic displacements associated with these modes are shown in Supplementary Table 1.

Two modes appear below *T*_c_ in the xy configuration at 77 cm^−1^ (9.5 meV) and 126 cm^−1^ (15.6 meV), labeled P_1_ and P_2_ in Fig. 1b, respectively. These modes were previously attributed to one-magnon excitations since they appear in the magnetically ordered state and have their largest intensity in xy (inset of Fig. 1b, Supplementary Fig. 2), indicating B_g_ symmetry^34^. Instead, the bulk magnon dispersion^35^ at 5 K shows a low-energy magnon at Γ below 1 meV (8 cm^−1^), similar to what was measured by recent FM resonance experiments^36^, and magnons at the M point of the Brillouin zone at ≈9 meV  and 15 meV. Furthermore, a recent Raman study of magnon excitations in FePS_3_ showed it is possible for magnons to be present in both xx and xy in quasi-2D van der Waals magnets^20^. Thus, the B_g_ nature of P_1_ and P_2_ is not conclusive evidence that they are magnons.

### Magneto-Raman measurements of 10 L CrI_3_ in Faraday geometry

**Fig. 2:**
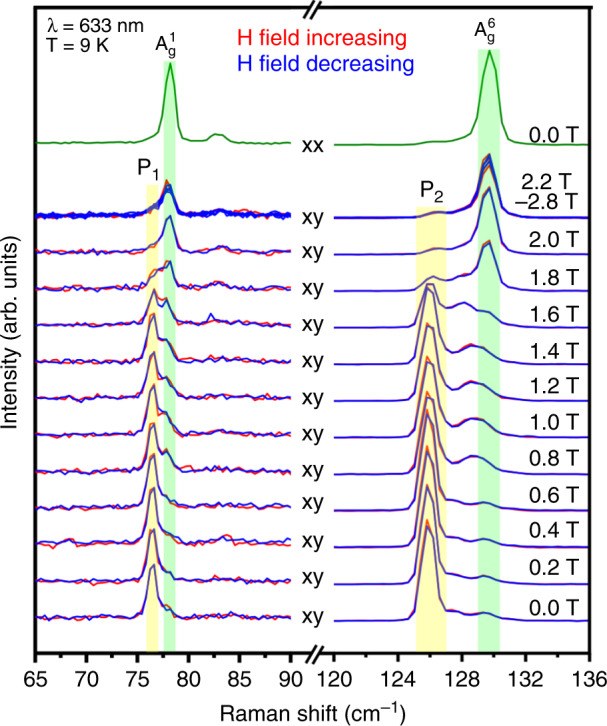
Magnetic field-dependent Raman spectra of CrI_3_. Spectra are shown from 0 T to 3 T (**B** ⊥ ab plane) at *T* = 9 K where P_1_ and P_2_ disappear above *B* = 2.0 T, whereas ${\mathrm{A}}_{\mathrm{g}}^1$ and ${\mathrm{A}}_{\mathrm{g}}^6$ appear despite being forbidden in the xy polarization configuration. Yellow (green) shading highlights changes in P_1_, P_2_ (${\mathrm{A}}_{\mathrm{g}}^1$, ${\mathrm{A}}_{\mathrm{g}}^6$) as the magnetic field is varied. A vertical offset was applied to spectra at different field values. xx spectrum at *B* = 0 T shown at top for comparison.

We studied the effects of an applied magnetic field perpendicular to the ab plane (**B** ⊥ ab, Faraday geometry) on P_1_ and P_2_, as detailed in Fig. 2. Two spectral ranges from 65 cm^−1^ to 90 cm^−1^ (P_1_, ${\mathrm{A}}_{\mathrm{g}}^1$) and 120 cm^−1^ to 136 cm^−1^ (P_2_, ${\mathrm{A}}_{\mathrm{g}}^6$) are shown on different intensity scales (≈a factor of 3:1, respectively) for clarity. At *B* = 0 T and in xy, P_1_ and P_2_ have strong intensities, whereas the two A_g_ modes at slightly higher frequencies have minimum intensities as they are forbidden in the xy configuration. Increasing the magnetic field results in drastic changes in the Raman spectra, where P_1_ and P_2_ behave in the same fashion. Above ≈1.6 T, the intensities of P_1_ and P_2_ abruptly start to vanish and ${\mathrm{A}}_{\mathrm{g}}^1$ and ${\mathrm{A}}_{\mathrm{g}}^6$ begin to appear in xy. By *B* = 2 T, P_1_ and P_2_ are absent in all polarization configurations, whereas ${\mathrm{A}}_{\mathrm{g}}^1$ and ${\mathrm{A}}_{\mathrm{g}}^6$ are no longer forbidden in xy. No further changes occur above 2 T, and no hysteresis was observed when the field was lowered back to 0 T. It should be noted that P_1_ and P_2_ do not show frequency shifting with magnetic field, suggesting they are not one-magnon processes with spins perpendicular to the ab plane.

**Fig. 3:**
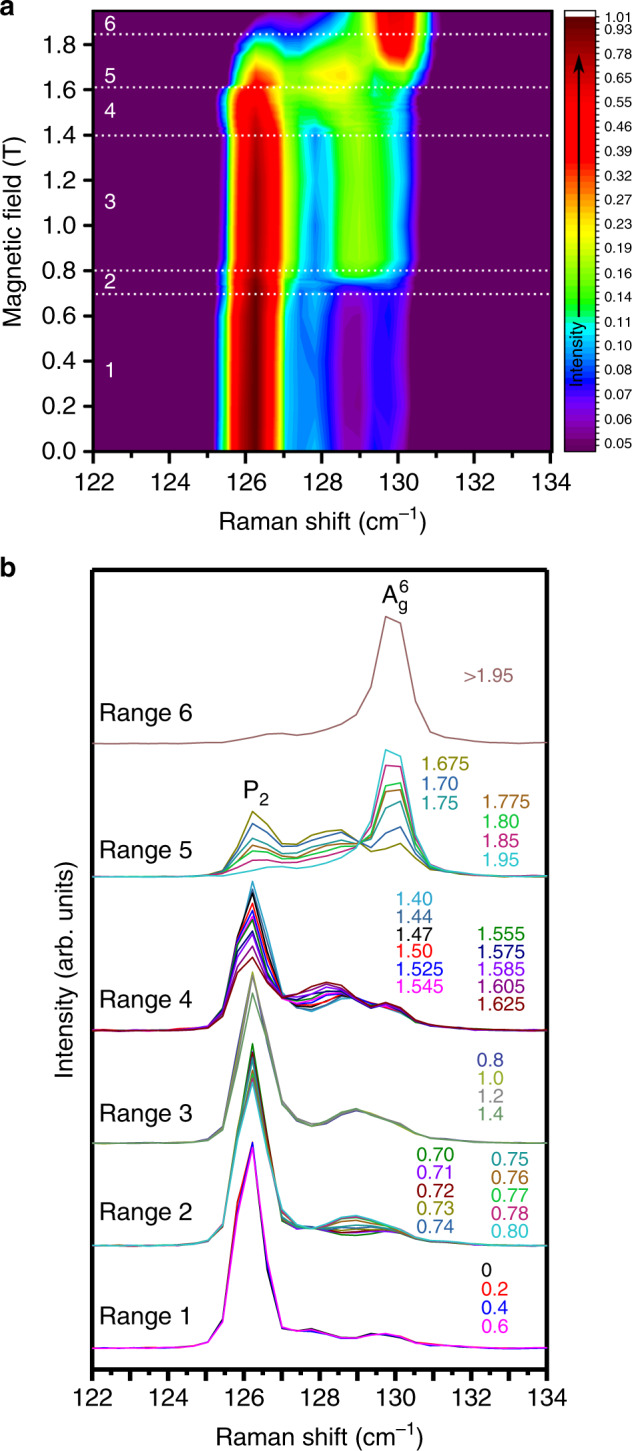
Distinct magnetic field ranges in CrI_3_. **a** False-color contour map of the Raman intensity (logarithmic) vs. magnetic field and Raman shift frequency. **b** Raman spectra at *T* = 9 K, showing a detailed view of the 122 cm ^−1^ to 134 cm^−1^ frequency range at different magnetic fields given by legend values (in Tesla). Six distinct field regions are identified below 2 T. A vertical offset was applied to spectra in the different field ranges for clarity.

Raman spectra were collected as the magnetic field was increased in finer steps up to 2 T. This is shown as a false-color map (Fig. 3a) and spectra (Fig. 3b) for the frequencies near P_2_ and ${\mathrm{A}}_{\mathrm{g}}^6$, where six distinct magnetic field ranges are revealed. Upon close inspection, there is additional Raman scattering intensity, i.e., spectral weight, present between P_2_ and ${\mathrm{A}}_{\mathrm{g}}^6$ ≈128 cm^−1^, although it cannot be discerned if the spectral weight is attributed to a single or multiple mode(s). No changes to P_2_, ${\mathrm{A}}_{\mathrm{g}}^6$, or the spectral weight were observed in Range 1 from 0 T and 0.6 T. In Range 2 between 0.7 T and 0.8 T, the spectral weight between P_2_ and ${\mathrm{A}}_{\mathrm{g}}^6$ appears to shift in frequency and intensity. The spectra are again stable through Region 3 from 0.8 T to 1.4 T, after which striking changes are seen in Regions 4 and 5. In Region 4, P_2_ starts to decrease in intensity, the intensity of ${\mathrm{A}}_{\mathrm{g}}^6$ stays relatively constant, and the spectral weight shifts in frequency and increases in intensity. In Region 5, P_2_ and the spectral weight both decrease in intensity until they disappear, whereas ${\mathrm{A}}_{\mathrm{g}}^6$ grows in intensity until *B* > 1.95 T (Region 6), when the phase transition is finally complete. It should be noted that experimental uncertainty, including instrumental drift and corrections for Faraday rotation in magneto-cryostat objective lenses, can lead to small changes in peak intensities (generally less than 5%) when comparing consecutively taken Raman spectra. The intensity changes being tracked in the field ranges in Fig. 3b, however, are more significant than any changes seen in the Γ point phonons (see Supplementary Fig. 3) and are thus outside of experimental uncertainty. Moreover, frequency shifts, such as those observed in the spectral weight between P_2_ and ${\mathrm{A}}_{\mathrm{g}}^6$ in Regions 2 and 4, are significantly more reliable than intensity changes, generally reproducible to within one CCD detector pixel (≈0.4 cm^−1^ with He–Ne excitation herein). For fitted peaks (e.g., well-defined phonons), changes in  the Raman shift frequency are even more precise (better than 0.1 cm^−1^).

Recent magneto-tunneling measurements of few-layered CrI_3_ observed large changes in the tunneling current at nearly the same magnetic field values where we observe marked changes in the Raman spectra, such as at 0.8 T and 2 T^12–15,25^. These changes were attributed to the spin-filtering effect when the magnetic field is strong enough to change the interlayer spin arrangement from AFM to FM, and the effect was observed even for 20 nm thick samples (our sample is ≈7 nm). The striking resemblance in the evolution with magnetic field observed with magneto-tunneling, including sharp changes between regions of stability, to those reported herein implies Raman spectroscopy is detecting the phase transition caused by layers flipping spins from AFM to FM stacking. The observation of the first jump at 0.8 T in a variety of thicknesses of CrI_3_ in magneto-tunneling^12–15,25^ suggests it is most likely a result of the surface layers (adjacent to the hBN) flipping while the second, final jump is the flipping of the internal layers at ≈2 T. This spin-flip phase transition is supported by the observation of the lower energy FM magnon for *B* > 6.5 T (Supplementary Fig. 4), which matches previous results on bulk, FM CrI_3_^35,36^, and the lack of change in the Raman spectra between 2 T and 9 T (Supplementary Fig. 5). The 10 L sample remains monoclinic at high magnetic fields (Supplementary Fig. 6), demonstrating that a change in crystal structure is not the source of the evolution in the Raman spectra. In particular, the spectral weight present between P_2_ and ${\mathrm{A}}_{\mathrm{g}}^6$ is extremely sensitive to the spin-flipping, displaying strong frequency shifts and intensity variations where the spin flips occur. The fact that the spectral weight does not shift in magnetic field in Regions 1 and 3, which are regions of stability, suggests its presence does not involve a one-magnon process. It is possible that the spectral weight results from the excitation laser wavelength (*λ* = 632.8 nm) being nearly resonant with the ligand-to-metal charge transfer transition^37–40^, but the weak Raman signal at off-resonance excitation laser wavelengths makes this difficult to verify.

**Fig. 4:**
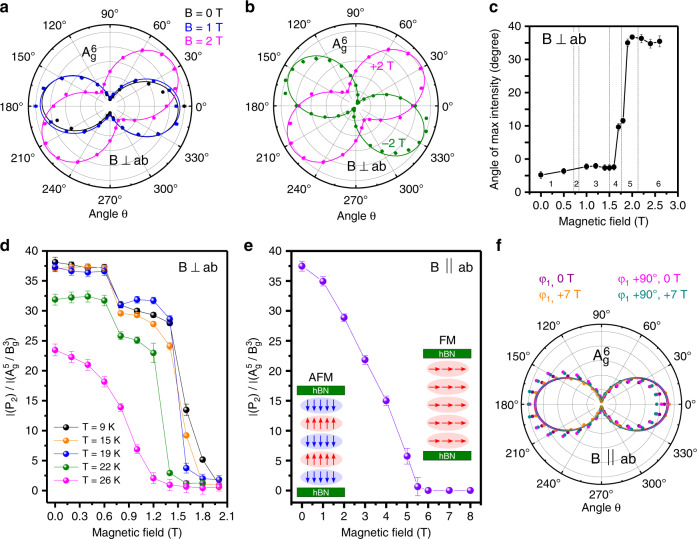
Observable changes in the intensities and angular dependence of the Raman modes with applied magnetic field. **a** Changes in the polar intensity of ${{\mathrm{A}}_{\mathrm{g}}^{6}}$ (maximum intensity normalized to one), as a function of *θ* for (a) *B* = 0 T (black), 1 T (blue), and 2 T (magenta) for **B** ⊥ ab plane. **b** Comparing the polar intensity of ${{\mathrm{A}}_{\mathrm{g}}^{6}}$ at positive (magenta) and negative (green) applied magnetic fields. **c** Angle of maximum intensity in polar plot in **a** as a function of magnetic field, where field ranges from Fig. 3b are marked. **d** Intensity of P_2_ relative to the intensity of the combined mode ${\mathrm{A}}_{\mathrm{g}}^5{\mathrm{/B}}_{\mathrm{g}}^3$, labeled $I\left( {{\mathrm{P}}_2} \right){\mathrm{/}}I\left( {{\mathrm{A}}_{\mathrm{g}}^5{\mathrm{/B}}_{\mathrm{g}}^3} \right)$, as a function of applied magnetic field (**B** ⊥ ab plane) at various sample temperatures. **e** $I\left( {{\mathrm{P}}_2} \right){\mathrm{/}}I\left( {{\mathrm{A}}_{\mathrm{g}}^5{\mathrm{/B}}_{\mathrm{g}}^3} \right)$ as a function of applied magnetic field (**B** ∥ ab plane). Insets of e show the magnetic stacking orders in the AFM and FM states for **B** ∥ ab plane. **f** Polar intensity of ${\mathrm{A}}_{\mathrm{g}}^6$ for a magnetic field (7 T) applied parallel to the ab plane at two different crystal orientations (*φ*_*1*_ and *φ*_*1*_ + 90°). Sample temperature was 9 K (2 K) for **B** ⊥ ab (**B** ∥ ab). Error bars represent standard errors from fitting function.

The polar plots for ${\mathrm{A}}_{\mathrm{g}}^6$, which track the intensity of ${\mathrm{A}}_{\mathrm{g}}^6$ as a function of angle *θ*, at various magnetic fields provide further evidence of the magnetic phase transition. As seen in Fig. 4a, the polar plot is rotated by ≈35° at *B* = 2 T when compared with *B* = 0 T. This can be understood if we write the Raman tensor for the A_g_ phonon with an induced magnetization *m* under applied magnetic field (**B** ⊥ ab) as

1 $$R_{A_{g,{\mathbf{B}} \bot {\mathrm{ab}}}} = R_{A_g,m = 0} + m_z \cdot R_{A_g,m_z}$$

which separates the spin-independent and spin-dependent terms. The point group of CrI_3_ requires that the total Raman tensor $R_{A_g}$ is symmetric under twofold rotation around the *b* axis (C_2x_). Thus, $R_{A_g,m = 0}$ and $m_z \cdot R_{A_g,m_z}$ must be symmetric under twofold rotation individually. As *m*_*z*_ itself is antisymmetric under C_2x_, then the form of $R_{A_g,m_z}$ is required to also be antisymmetric under C_2x_ such that their product is symmetric:

2 $$R_{A_g,{\mathbf{B}} \bot {\mathrm{ab}}} = \left( {\begin{array}{*{20}{c}} \alpha & 0 \\ 0 & \beta \end{array}} \right) + m_z\left( {\begin{array}{*{20}{c}} 0 & \gamma \\ \delta & 0 \end{array}} \right)$$

Thus, for the *B* = 0 T case (AFM state) where there is no induced magnetization, the Raman tensor would be purely symmetric, and we would not expect to detect the A_g_ phonon in xy polarization configurations. A magnetic field applied perpendicular to the ab plane that spin-polarizes the CrI_3_ to the FM state, however, introduces off-diagonal tensor elements and breaks this expectation, causing the polar plot to rotate as seen in Fig. 4a. Assuming there are minimal changes between the monoclinic (C_2h_), 10 L CrI_3_ and the hexagonal point group of the monolayer (D_3d_), then *β* ≈ *α* + Δ and *δ* ≈ −(*γ* + Δ′), where Δ and Δ′ are small corrections.

3 $$R_{A_{g}, + B_{z}} = \left( {\begin{array}{*{20}{c}} \alpha & {m_{z}\gamma} \\ { - m_{z}(\gamma + \Delta ^\prime )} & {\alpha + \Delta } \end{array}} \right),\\ R_{A_{g}, - B_{z}} = \left( {\begin{array}{*{20}{c}} \alpha & { - m_{z}{\gamma}} \\ {m_{z}(\gamma + \Delta ^\prime )} & {\alpha + \Delta } \end{array}} \right)$$

Writing the matrices as in Eq. (3) makes it clear that when the magnetic field direction is flipped from **B**_**z**_ to −**B**_**z**_, thus flipping the direction of the induced magnetization, the signs of the off-diagonal elements switch, resulting in the opposite rotation of the polar plot, exactly as we observed in Fig. 4b and Supplementary Fig. 7.

Figure 4c shows the angle of maximum intensity of the polar plot in 4a as the magnetic field is swept through the phase transition, where Regions 1–6 from Fig. 3 are marked with vertical dashed lines. Although Fig. 3 and magneto-tunneling results indicate that parts of the phase transition occur between Regions 2 and 4, there is no observable change in the maximum intensity of the polar plot in those field ranges. In Region 5, however, it increases to ≈10° for *B* = 1.7 T and 1.8 T, and then finally to ≈35° for 1.8 T and above. The lack of rotation of the polar plot between 0.7 T and 0.8 T reveals that not enough magnetization is induced in this field range to introduce the off-diagonal Raman tensors in Eq. (2).

The temperature dependence of the spin flips was investigated by tracking the intensity of P_2_ as a function of magnetic field for different temperatures between *T* = 9 K and 26 K, as detailed in Fig. 4d. The intensity of P_2_ is shown relative to the intensity of the combination peak ${\mathrm{A}}_{\mathrm{g}}^5{\mathrm{/B}}_{\mathrm{g}}^3$ at ≈115 cm^−1^ at *B* = 0 T for each temperature, as this peak appears to remain constant with temperature and magnetic field (in the probed range). The spin-flip transition field, or the amount of magnetic field necessary to cause P_2_ to disappear, decreases with increasing temperature. In addition, the distinct jumps from spin flips and flat plateaus observed in the intensity of P_2_ smooth out for higher *T*. Further temperature dependence is analyzed in Supplementary Fig. 8. This behavior is consistent with a phase transition where there is a strong correlation between the temperature and magnetic field, which suggests that the magnetic field takes the transition temperature to zero. Although this behavior is akin to a quantum phase transition, there is no evidence yet of any quantum critical behavior in this material, but it is a very interesting avenue for future investigation.

### Magneto-Raman measurements of 10 L CrI_3_ in Voigt geometry

Finally, we investigated the directional dependence of the spin-flip phase transition by rotating the sample such that the applied magnetic field is parallel to the ab plane (**B** ∥ ab, Voigt geometry). Figure 4e shows $I\left( {{\mathrm{P}}_2} \right){\mathrm{/}}I\left( {{\mathrm{A}}_{\mathrm{g}}^5{\mathrm{/B}}_{\mathrm{g}}^3} \right)$ for **B** ∥ ab and at *T* = 2 K. Unlike in the case for **B** ⊥ ab, no jumps or plateaus are observed in the intensity of P_2_. Instead, the intensity of P_2_ continuously decreases with increasing applied magnetic field up to *B* = 6 T, after which the layers are stacked FM with the spins pointing in the ab plane. This observation matches nicely with measurements of the magneto-tunneling current when the field is in the ab plane^12–15^. Furthermore, no frequency shift of P_2_ is observed as a function of magnetic field (Supplementary Fig. 9). We perform the same tensor analysis that was done for Eqs. (1) and (2) above, but this time for the magnetic field applied in the ab plane.

4 $$R_{A_g,{\mathbf{B}}\parallel {\mathrm{ab}}} = R_{A_g,m = 0} + m_x \cdot R_{A_g,m_x} + m_y \cdot R_{A_g,m_y}$$

where *x* (*y*) corresponds to *b* axis (*a* axis). Requiring that the total Raman tensor be symmetric under C_2x_, then $R_{A_g,{\mathbf{B}}\parallel {\mathrm{ab}}}$ is written as:

5 $$R_{A_g,{\mathbf{B}}\parallel {\mathrm{ab}}} = R_{A_g,m = 0} + m_x\left( {\begin{array}{*{20}{c}} {\alpha ^\prime } & 0 \\ 0 & {\beta ^\prime } \end{array}} \right) + m_y\left( {\begin{array}{*{20}{c}} 0 & {\gamma ^\prime } \\ {\delta ^\prime } & 0 \end{array}} \right)$$

From Eq. (5), when the magnetic field is directed along the *b* axis and strong enough to align the spins and create a net magnetization *m*_*x*_, we expect the Raman tensor to be symmetric and the polar plot of the intensity of ${\mathrm{A}}_{\mathrm{g}}^6$ as a function of *θ* not to rotate. However, when the magnetic field has a component pointed along the *a* axis that results in a net magnetization *m*_*y*_, off-diagonal tensor elements are introduced, and some rotation of the polar plot would be expected. To test these predictions, we rotated the sample while the magnetic field was applied in the ab plane such that the magnetic field was pointed along two different crystal orientations *φ*_*1*_ and *φ*_*2*_ = *φ*_*1*_ + 90°. The polar plot of the intensity of ${\mathrm{A}}_{\mathrm{g}}^6$ at *B* = 0 T and 7 T (in the spin-polarized state) are shown in Fig. 4f. Interestingly, for both orientations, in which either *φ*_*1*_ or *φ*_*2*_ must contain magnetic field components along the *a* axis, the polar plot of the intensity of ${\mathrm{A}}_{\mathrm{g}}^6$ does not show the same rotation that was observed for spins pointing perpendicular to the ab plane in the spin-polarized state. This indicates that the off-diagonal elements *γ*′ and *δ*′ are negligible for magnetic fields pointing in the ab plane.

## Discussion

**Fig. 5:**
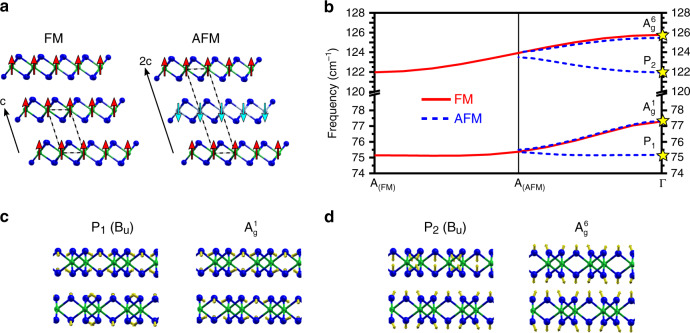
Unit cells, phonon dispersions, and normal modes for FM and AFM stacking. **a** Comparing the unit cell for the FM and AFM stacked structures, where the AFM unit cell is doubled in the *c* direction. Chromium atoms are green, whereas iodine atoms are blue. **b** Calculated phonon dispersion showing the Raman-active modes in the FM (red, solid) and AFM (blue, dashed) stacking in bulk CrI_3_ in the monoclinic crystal structure. In the AFM case, the doubling of the unit cell along the *c* direction in real space results in the A_(FM)_-point folding into Γ, such that the number of modes is doubled compared with the FM case. The Γ–A distance in the AFM case is half that of the FM case. Modes that correspond to P_1_, ${\mathrm{A}}_{\mathrm{g}}^1$, P_2_, and ${\mathrm{A}}_{\mathrm{g}}^6$ are highlighted and their atomic vibrations are shown in **c** and **d**.

Our data shows that P_1_ and P_2_, which appear (disappear) in the AFM (FM) state and are absent in bulk samples (Supplementary Fig. 10), are not one-magnon excitations, as their frequencies do not shift with applied magnetic field. After considering other theoretical models (see Supplementary Notes 1 and 2, Supplementary Tables 2–5, Supplementary Fig. 11), we conclude that P_1_ and P_2_ are actually zone-folded phonons owing to a doubling of the AFM unit cell in the *c* direction. This is illustrated in Fig. 5a, where the opposite spins of consecutive layers in the AFM configuration lead to a unit cell that is twice as large in the *c* direction compared to the FM configuration.

Using DFT, we calculated the phonon dispersions for monoclinic, bulk CrI_3_ in both the FM and AFM stacking configurations. The full phonon dispersions, Brillouin zone, and table of frequencies are given in Supplementary Figs. 12 and 13 and Supplementary Table 6. Figure 5b shows only the Raman-active phonons in the FM state (red, solid) and AFM state (blue, dotted) for frequencies near P_1_ and P_2_. In general, Raman spectroscopy is only sensitive to modes at the Γ point in the phonon dispersion due to conservation of momentum. Thus, phonons at the A point (in direction of **k**_**z**_) in the FM state are not observed. However, the doubling of the unit cell in the AFM state leads to zone-folding, where the phonons at the A point in the Brillouin zone in the FM state fold back onto Γ in the AFM state and can be observed in Raman spectroscopy. Two pairs of modes with similar frequencies to P_1_/${\mathrm{A}}_{\mathrm{g}}^1$ and P_2_/${\mathrm{A}}_{\mathrm{g}}^6$ have an easily resolvable frequency splitting at Γ (highlighted Fig. 5b), with the zone-folded phonon (P_1_ or P_2_) between 2 cm ^−1^ and 4 cm^−1^ lower in frequency than the original phonon (${\mathrm{A}}_{\mathrm{g}}^1$ or ${\mathrm{A}}_{\mathrm{g}}^6$). Illustrations of these vibrations are shown in Fig. 5c, d, where the two layers vibrate in-phase for ${\mathrm{A}}_{\mathrm{g}}^1$ and ${\mathrm{A}}_{\mathrm{g}}^6$ and out-of-phase for P_1_ and P_2_. In the bulk, P_1_ and P_2_ would have B_u_ symmetry and would thus be Raman silent but infrared active. However, the breaking of inversion symmetry for an even number of layers in the AFM configuration would allow P_1_ and P_2_ to be Raman active with B symmetry (only seen in xy configurations). These zone-folded phonons would not shift in magnetic field and would be Raman-active (forbidden) in the AFM (FM) state, aligning with the observed behaviors of P_1_ and P_2_.

Even though the doubling of the unit cell is purely magnetic in nature, the observation of very strong zone-folded phonons that have similar intensity as the observed Γ point phonons reveals the strong coupling between magnetism and the lattice in atomically thin CrI_3_. Of note, the origins of P_1_ and P_2_ as zone-folded phonons predict they would disappear for a monolayer sample (no zone-folding) or a sample with an odd number of layers because the AFM state preserves inversion symmetry with an odd number of layers. Recent work^41,42^ has shown P_1_ and P_2_ are not present in monolayers of CrI_3_, yet data by Jin et al.^34^ suggests the presence of P_1_ and P_2_ for odd layer thicknesses. One possibility is that the encapsulation of thin layers of CrI_3_ in hBN breaks inversion symmetry naturally, leading to the presence of P_1_ and P_2_ in all thicknesses. Further studies as a function of layer thickness and encapsulation parameters are needed to elucidate the symmetry response of P_1_ and P_2_ for even vs. odd numbers of layers.

In conclusion, we utilized temperature-dependent, magneto-Raman spectroscopy to elucidate a magnetic phase transition in CrI_3_ where the interlayer stacking changes from AFM to FM. Substantial changes in the Raman spectra are detected at specific magnetic field values due to spin flips of layers to a FM stacking state, indicating that Raman modes are extremely sensitive to this phase transition. Moreover, Raman scattering proves to be crucial to understanding the symmetry and frequency shifts of the modes. We deduce that the modes P_1_ and P_2_ are not high frequency magnons as previously concluded, but instead attribute the modes to zone-folded phonons using symmetry arguments, polarization-dependent Raman responses, and calculated phonon dispersions in the FM and AFM stacking configurations. This study paves the way for further use of magneto-Raman spectroscopy to investigate spin-flip phase transitions in 2D van der Waals magnets.

## Methods

### Sample preparation and encapsulation

Bulk CrI_3_ crystals were grown by a chemical vapor transport technique using stoichiometric mixtures of Cr and I in a sealed evacuated quartz tube, as mentioned in other references^5,24^. The phase of the obtained crystals were checked by X-ray diffraction. These crystals were then exfoliated onto Si/SiO_2_ substrates in an Ar-filled glovebox having O_2_ and H_2_O concentrations of <0.1 ppm. hBN/CrI_3_/hBN heterostructures were fabricated using a dry transfer technique detailed elsewhere^23,43^. Specifically, PDMS (polydimethylsiloxane) was used as the polymer stamp.

### Raman spectroscopy

A triple grating Raman spectrometer (Horiba JY T64000^†^, 1800 mm^−1^ grating) coupled to a liquid nitrogen cooled CCD detector was used to collect Raman spectra. The excitation wavelength was 632.8601 nm from a He–Ne laser and spectra were measured in the 180° backscattering configuration. Raman spectra as a function of temperature and magnetic field were collected using an attoDRY cryostat (Attocube Inc.^†^), where the sample was zero-field cooled and studied with a magnetic field compatible objective (×50, N.A. 0.82). Ultra-broadband polarizers and achromatic half wave plates were used to select and control polarization, including correcting for Faraday rotation in the objective under applied magnetic field. The laser power was ≈150 µW to avoid local heating and integration times were ≈12 min.

### DFT phonon calculations

We performed DFT calculations^44,45^ with the Quantum Espresso^46^ code, using the GBRV ultrasoft pseudopotential set^47,48^. We used the vdw-df-ob86 exchange correlation functional^49,50^, which includes long range van der Waals interactions, for our main results. We also tested the PBEsol^51^ functional, finding similar results. Phonon calculations were performed using a finite differences (frozen phonon) approach, using PHONONPY^52^ to perform symmetry analysis and the cluster_spring^53^ code to calculate phonon dispersions. A 6 × 6 × 4 k-point sampling was used for the ferromagnetic unit cell.

(Certain commercial equipment, instruments, or materials are identified in this manuscript in order to specify the experimental procedure adequately. Such identification is not intended to imply recommendation or endorsement by the National Institute of Standards and Technology, nor is it intended to imply that the materials or equipment are necessarily the best available for the purpose).

## Supplementary information

Supplementary Information

## Data Availability

The data that support the findings of this study are available from the corresponding author upon reasonable request.
