# Supplementary material for: Distinct magneto-Raman signatures of spin-flip phase transitions in CrI3
Source: Nat Commun. 2020 Aug 3;11:3879. doi: 10.1038/s41467-020-17320-3 (PMC7398929; doi:10.1038/s41467-020-17320-3)
Supplement: Supplementary file 1 — Supplementary Information [file 41467_2020_17320_MOESM1_ESM.pdf]

SUPPLEMENTARY INFORMATION

Distinct magneto-Raman signatures of spin-flip phase transitions in CrI_3

McCreary *et al.*

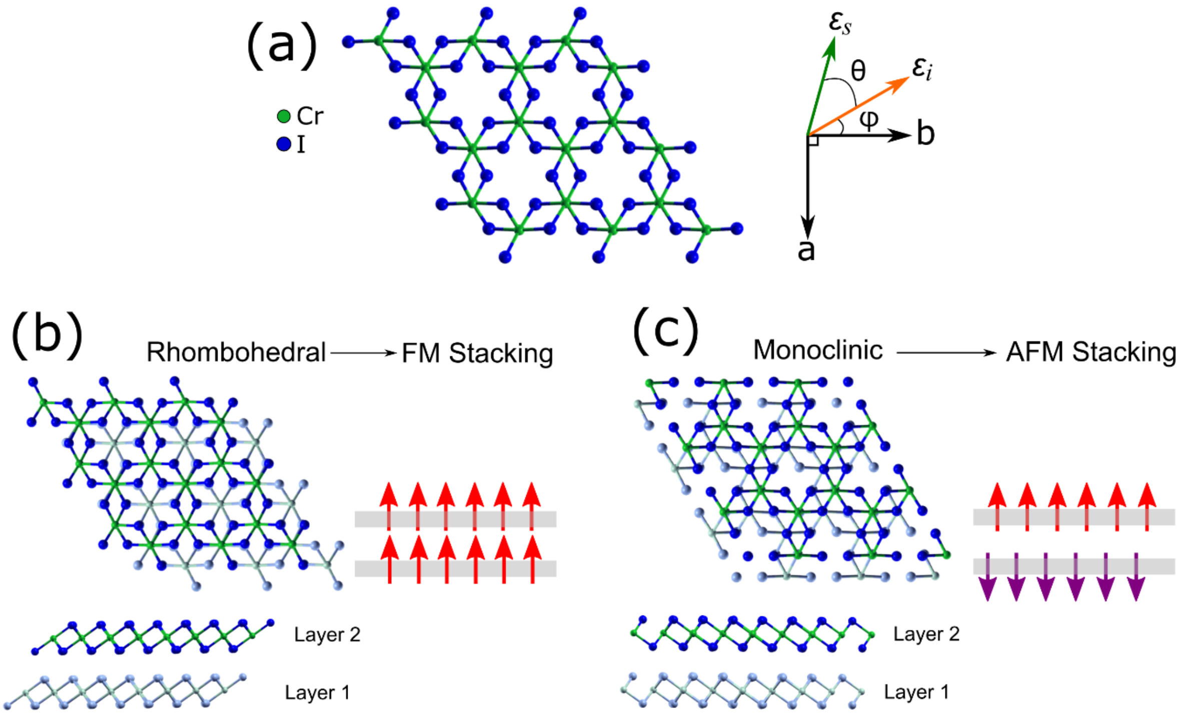

Supplementary Figure 1: (a) Schematic showing top view of the crystal structure of a monolayer of CrI_3. (b,c) Comparing between the (b) rhombohedral and (c) monoclinic stacking of two layers of CrI_3, where the rhombohedral (monoclinic) results in ferromagnetic (antiferromagnetic) spin stacking.

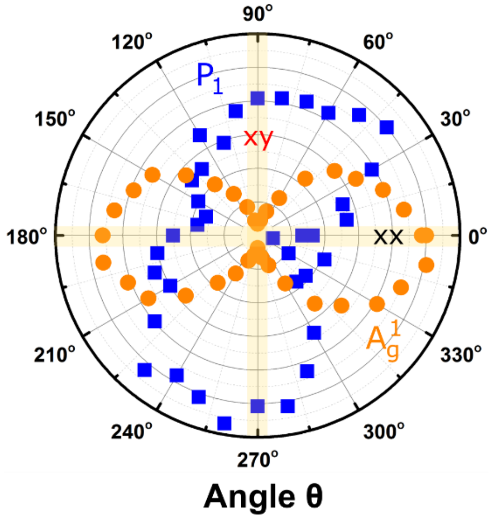

Supplementary Figure 2: Polar intensity plot as a function of θ for P_1 mode and nearby A_g^1 phonon at $T = 5$ K and $B = 0$ T.

**Frequency (cm^{-1})**	**Symmetry**	**Vibration**
51.09	B_g	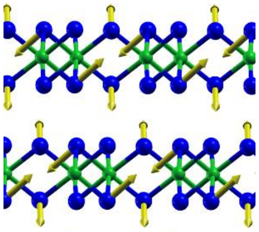
52.15	A_g	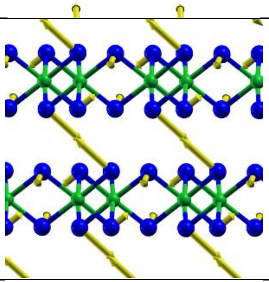
77.00	A_g	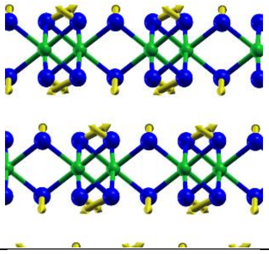
87.92	B_g	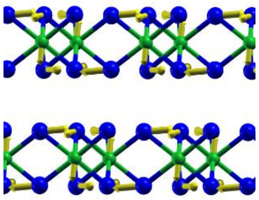
100.99	A_g	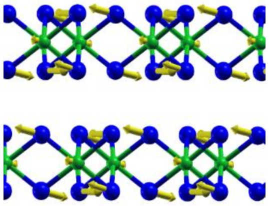
101.22	B_g	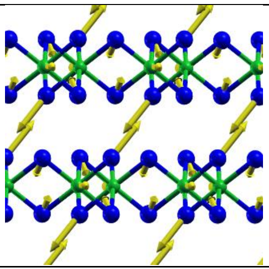
Continued…		

102.54	B_g	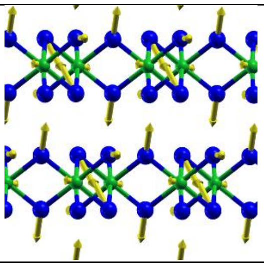
103.77	A_g	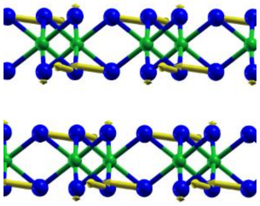
125.94	A_g	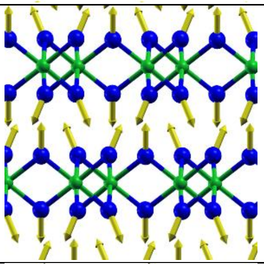
202.74	B_g	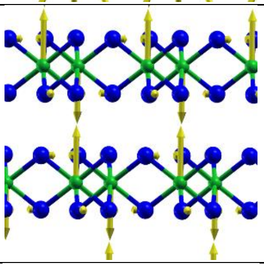
226.30	A_g	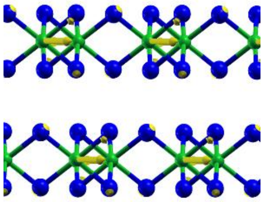
227.93	B_g	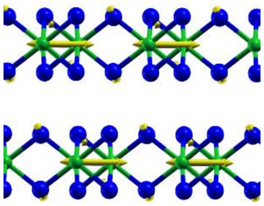

Supplementary Table 1: DFT-calculated Raman-active phonons in monoclinic, bulk CrI_3.

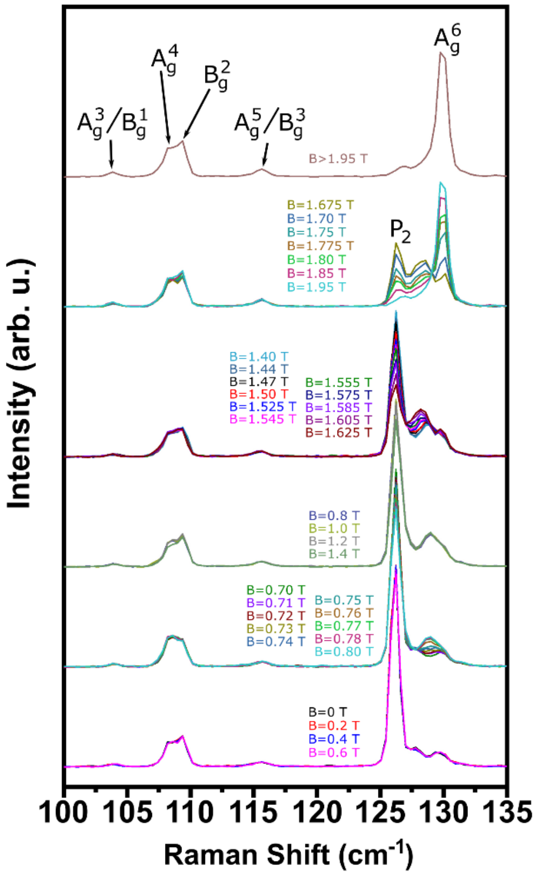

Supplementary Figure 3: Raman spectra of 10 L CrI_3 as a function of magnetic field (applied perpendicular to the ab plane) at $T = 9$ K, showing a larger frequency range compared with Figure 3 of the main text where negligible changes are observed in the other phonon modes A_g^3/B_g^1, A_g^4, B_g^2, and A_g^5/B_g^3.

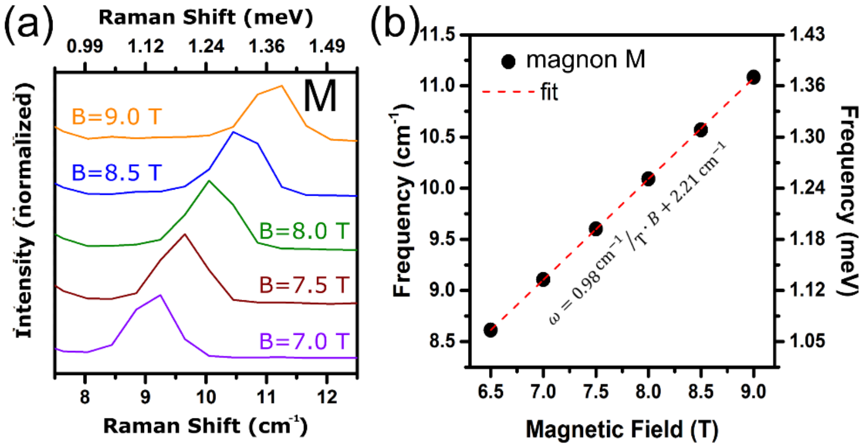

Supplementary Figure 4: (a) Low-frequency Raman spectra at T = 5 K showing the true FM resonance (FMR), *i.e.* magnon, in CrI_3 that blueshifts with increasing magnetic field. At lower field values, we are unable to observe the FMR since it is below our spectrometer cutoff (~ 7 cm^{-1}). From the fit in (b), we extract $g \approx 2.08 \pm 0.05$ and $\omega_{B=0T}$ = 2.21 ± 0.3 cm^{-1} (0.27 ± 0.04 meV).

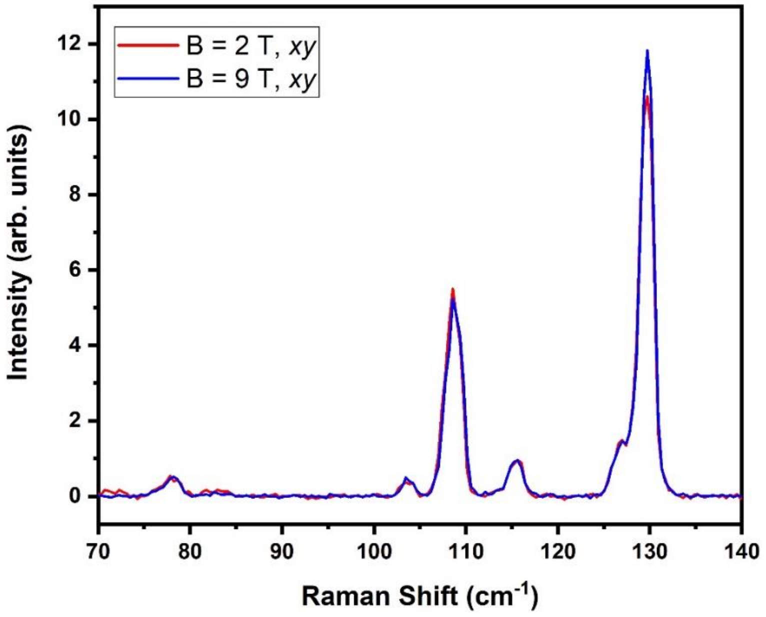

Supplementary Figure 5: Comparing the Raman spectra (T = 9 K) of 10 L CrI_3 at B = 2 T and 9 T, where the magnetic field is applied perpendicular to the ab plane (*i.e.* parallel/antiparallel to the direction of the spins). Negligible changes are observed between the two spectra.

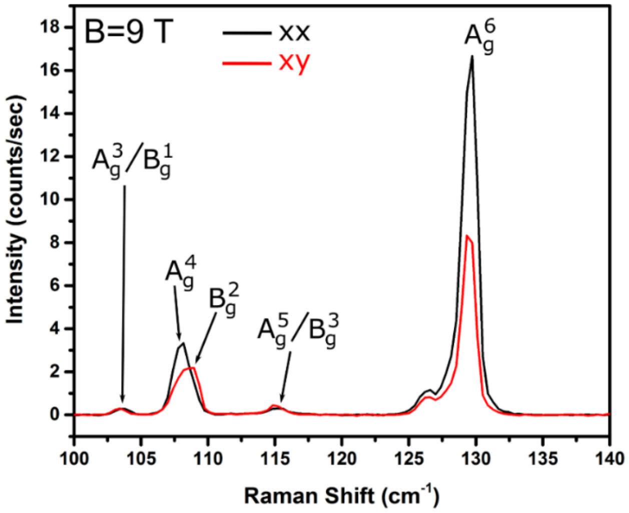

Supplementary Figure 6: Comparing Raman spectra at B = 9 T (**B** ⊥ ab) and T = 9 K in the parallel (xx, black) and cross (xy, red) polarization configurations. Since we still observe a splitting in the mode at ≈108 cm^{-1}, as opposed to one degenerate mode, we deduce the thin CrI_3 is still in the monoclinic phase after the magnetic phase transition from AFM to FM interlayer stacking.

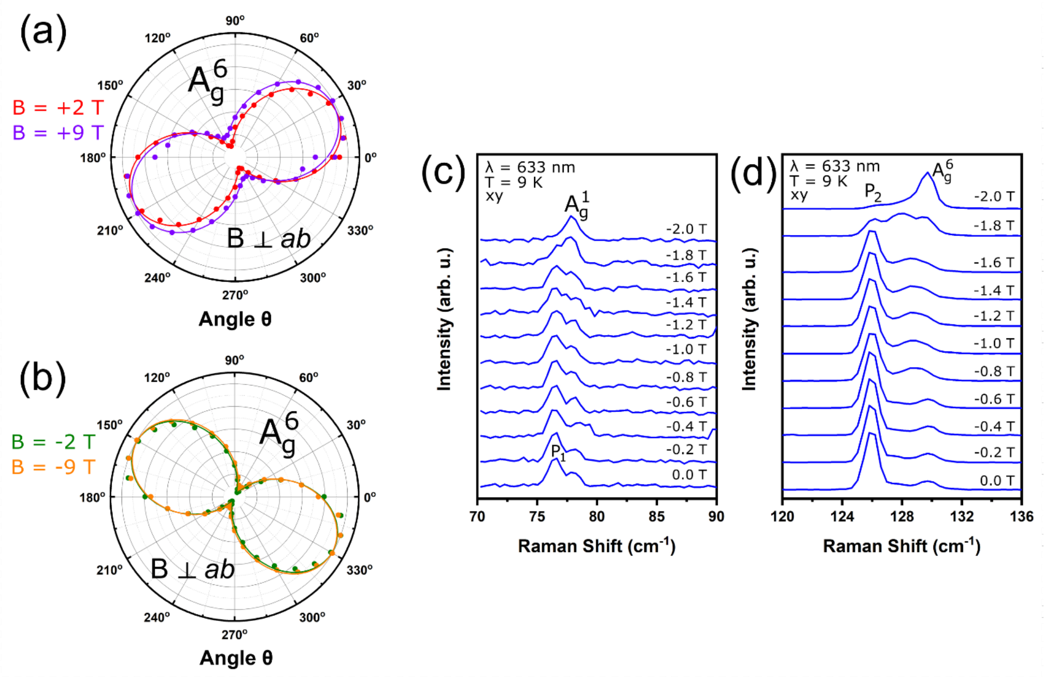

Supplementary Figure 7: Comparing polar intensity plots for A_g^6 for (a) B = +2 T and +9 T and (b) B = -2 T and -9 T at 9 K, with the magnetic field applied perpendicular to the ab plane. Frequency range showing (c) P_1, A_g^1 and (d) P_2, A_g^6 as a function of applied negative magnetic field (**B** ⊥ ab), showing the same trend as applied positive magnetic fields.

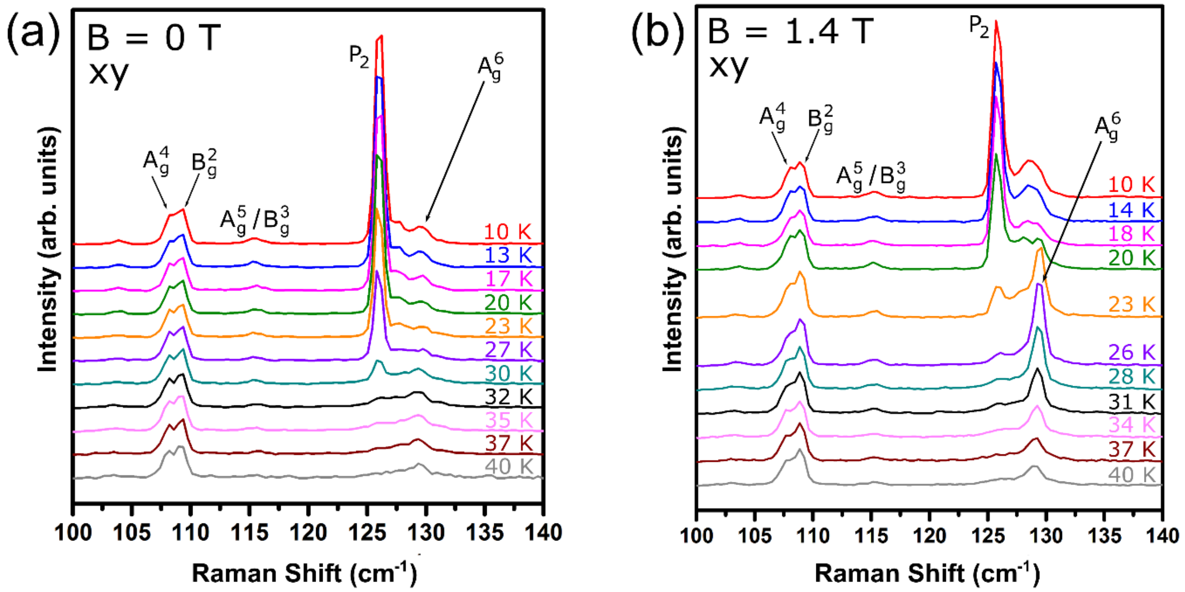

Supplementary Figure 8: (a) Temperature dependence of the Raman spectra at B = 0 T in xy polarization configuration, where P_2 disappears above 32 K and the A_g^6 mode remains small. (b) Temperature dependence at B = 1.4 T, where increasing the temperature results in a similar evolution of the Raman spectra as further application of magnetic field, including the appearance/increased intensity of A_g^6, until it disappears again as the sample is warmed (**B** ⊥ ab). Supplementary Figures 8a and 8b demonstrate that increasing temperature can have the same effect as increasing magnetic field to complete the phase transition in thin CrI_3. Supplementary Figure 8a shows the evolution of the Raman spectra at B = 0 T, in xy configuration, as a function of temperature. As the temperature of the CrI_3 is increased, the intensity of P_2 decreases until it is unobservable above T = 35 K. In this case, with no magnetic field applied, the intensity of A_g^6 remains small, as expected. On the contrary, when a magnetic field slightly below B_C is applied, such as B = 1.4 T in Supplementary Figure 8b, increasing the temperature can be used in the same fashion as increasing the magnetic field to push CrI_3 through the phase transition, as was shown in magneto-resistance as well.[1] In this case, increasing the temperature by ≈ 16 K is enough to drive the phase transition that would have required an extra ≈ 0.6 T of magnetic field. With the magnetic field applied, the intensity of A_g^6 increases with increasing temperature, but as the temperature increases further towards the Curie temperature, A_g^6 starts to disappear again, confirming that the phase transition we are observing is magnetic in nature.

*In Supplementary Figure 8b, the sample was zero field-cooled to T = 10 K, where a magnetic field of 1.4 T was applied perpendicular to the ab plane. Keeping the field at 1.4 T, the temperature was then increased to the values shown in the graph.

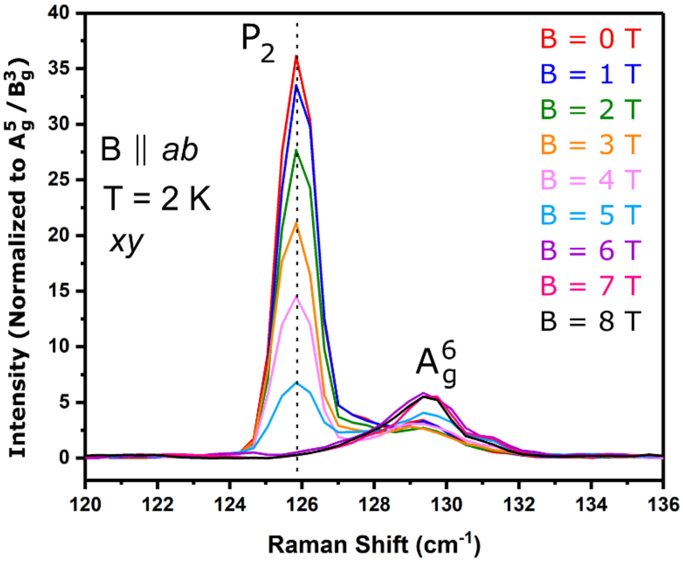

Supplementary Figure 9: Evolution of P_2 and A_g^6 as a function of applied magnetic field when the field is applied parallel to the ab plane. Unlike the case of **B** ⊥ ab, a continuous decrease of P_2 as a function of field is observed. No frequency shift of P_2 is seen.

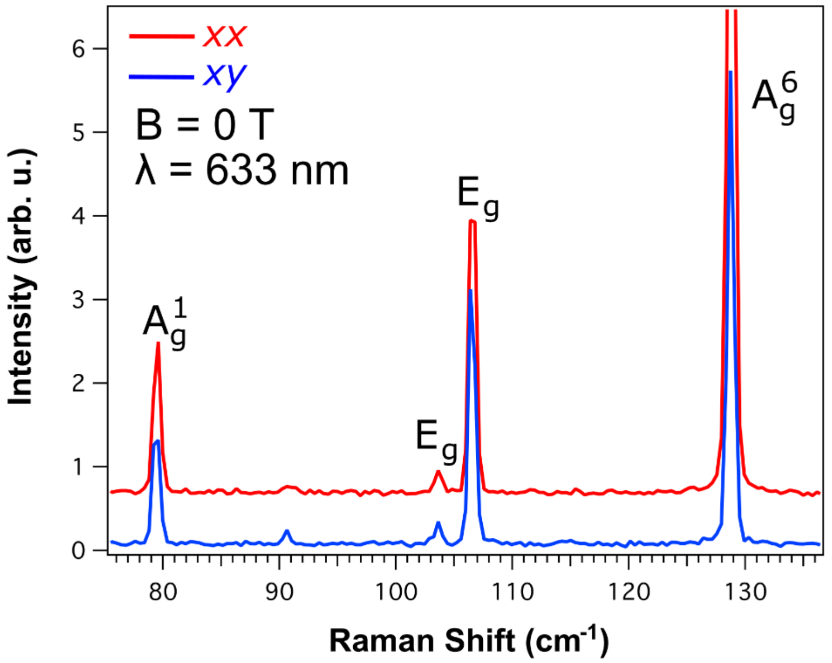

Supplementary Figure 10: Raman spectra for bulk (≈1 mm thickness, not encapsulated in hBN) CrI_3 at T = 5 K (**B** ⊥ ab). The phonons are labeled using the rhombohedral point group $3\overline{R}$, since bulk CrI_3 does undergo a phase transition from monoclinic to rhombohedral at ≈ 200 K. Modes P_1 and P_2 were not observed, and in agreement with the rhombohedral structure, the mode at ≈ 107 cm^{-1} is degenerate in xx and xy polarization configurations.

Supplementary Note 1: $\mathbf{P_1}$ and $\mathbf{P_2}$ as Bound Two-Magnon Excitations

We considered the possibility that $\mathrm{P_1}$ and $\mathrm{P_2}$ could be intralayer bound two-magnon excitations with a total spin of zero, given that they do not shift with *B* field. A two-excitation consists of one magnon propagating in the $+\boldsymbol{k}$ direction and other magnon propagating in the $-\boldsymbol{k}$, such that $+\boldsymbol{k}-\boldsymbol{k}=0$ and conservation of momentum is conserved, allowing these excitiations to be seen in Raman spectroscopy. If the bound two-magnon excitation had a total spin of zero, then the excitation would not be expected to shift in magnetic field, as was experimentally observed for $\mathrm{P_{1,2}}$. In ferromagnetic systems, two-magnon excitations are expected to naturally arise from bond-dependent interactions like the Kitaev interaction,[2] which a recent experiment[3] suggests is the dominant interaction of CrI_3. We can see how the Kitaev interaction can produce two-magnon excitations by expressing the Kitaev interaction in the Holstein-Primakoff representation, which yields terms of the form $a_{\boldsymbol{k}}^{\dagger}a_{-\boldsymbol{k}}^{\dagger}$, corresponding to the creation of a pair of magnons with opposite momenta.

We performed an exact diagonalization calculation of the two-magnon density of states (DOS) and Raman intensity at zero temperature for a spin-3/2 system of 6-sites (a single honeycomb plaquette) described by the *JKΓ* Hamiltonian

$$\hat{H}_{JK\Gamma}=\sum_{\langle \boldsymbol{r},\boldsymbol{r}'\rangle\in\alpha\beta(\gamma)}\left[J\boldsymbol{S}_{\boldsymbol{r}}\cdot\boldsymbol{S}_{\boldsymbol{r}'}+KS_{\boldsymbol{r}}^{\gamma}S_{\boldsymbol{r}'}^{\gamma}+\Gamma\left(S_{\boldsymbol{r}}^{\alpha}S_{\boldsymbol{r}'}^{\beta}+S_{\boldsymbol{r}}^{\beta}S_{\boldsymbol{r}'}^{\alpha}\right)\right] \quad (1)$$

which is the most general nearest-neighbor spin-spin interaction Hamiltonian allowed by the symmetries of a CrI_3 monolayer, where $\gamma\in\{x,y,z\}$ labels the bond type through which the neighboring Cr ions at $\boldsymbol{r},\boldsymbol{r}'$ are interacting, α,β label the other two bond types, J is the Heisenberg coupling, K is the Kitaev coupling, and Γ is the symmetric off-diagonal coupling. Using the values of the coupling constants obtained by Lee *et al.*,[3] namely $J=-0.212$ meV, $K=-5.190$ meV, and $\Gamma=-0.068$ meV, we found that the entire two-magnon DOS and Raman spectrum (see Supplementary Figure 12) shift with applied *B* field, thereby ruling out this potential mechanism for $\mathrm{P_1}$ and $\mathrm{P_2}$.

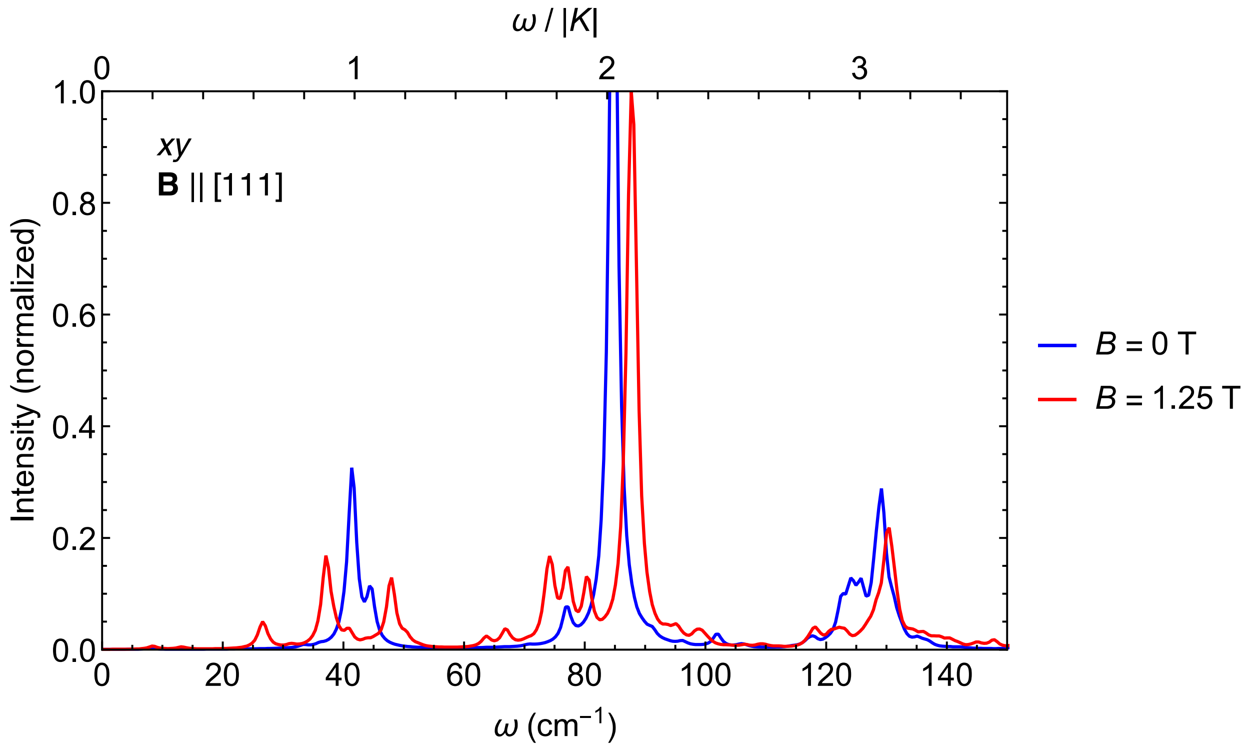

Supplementary Figure 11: Exact diagonalization calculation of the zero-temperature Raman spectra in the cross (xy) polarization configuration for a spin-3/2 system of 6-sites (a single honeycomb plaquette) described by the *JK*Γ Hamiltonian and using the values of the coupling constants obtained by Lee *et al*.[3] This calculation serves as an estimate of the Raman behavior of monolayer CrI_3. When a magnetic field is applied out of the plane, the entire Raman spectrum shifts.

Supplementary Note 2: P_1 and P_2 as Bound Magnon-Phonon Pairs

Another theoretical model we considered was that if the spins in CrI_3 were oriented in-plane and antiferromagnetically stacked, as has been reported in $CrCl_3$,[4] P_1 and P_2 could correspond to the bound state of a phonon and a magnon, where the phonon would be at a slightly higher frequency (A_g^1 for P_1, and A_g^6 for P_2) and the magnon would be a soft magnon $\gamma_{(0,0,\pi),+}$. We considered this as an option because the magnon (and hence the phonon-magnon bound state) carries B_g representation of the $2'/m$ magnetic group in our 10-layer sample and exhibits the highest intensity in cross polarization, as was observed for $P_{1,2}$. The associated magnetic group $2'/\mathrm{m}$ generated by magnetic rotation C'_{2x} and mirror M_x for the spins oriented in the ab plane is also consistent with the angle-dependence of second harmonic generation (SHG) signals observed by Z. Sun *et al.*,[5] since mirror M_x and 2-fold rotation C_{2x} enforces similar constrains to nonlinear susceptibility with respect to in-plane polarization. When the perpendicular magnetic field B_z exceeds the critical value of 2 T, the in-plane order AFM_x could be destabilized and transitioned into out-of-plane ferromagnetism FM_z at larger fields.

We use the isotropic Heisenberg interaction between nearest neighbors as a minimal model in order to extract the symmetry properties of the magnon modes. The acquired representation of magnon modes and selection rules from Raman scattering are expected to be universal, insensitive to the exact form of the Hamiltonian. However, the frequency of the magnon modes can be an artifact of such toy models and should not be compared directly with experiments.

We label the lattice sites of the layered honeycomb lattice by

$$\boldsymbol{R} = \boldsymbol{r} + z\vec{a}_3 \equiv (r_1, r_2, z, s), \quad s = A/B \text{ labeling two sublattices} \tag{2}$$

$$\boldsymbol{r} \equiv r_1\vec{a}_1 + r_2\vec{a}_2 + \vec{R}_s, \quad r_{1,2}, z \in \mathbb{Z} \tag{3}$$

where $\{\vec{a}_i | i = 1,2,3\}$ are the primitive lattice vectors, and $\boldsymbol{r}$ is the in-plane 2D coordinate.

The monoclinic space group $2/\mathrm{m}$ is generated by the following point group symmetries:

$$(x, y, z) \xrightarrow{M_x} (-x, y, z) \tag{4}$$

$$(x, y, z) \xrightarrow{C_{2x}} (x, -y, -z) \tag{5}$$

and their combination is the inversion symmetry.

The following minimal model of spin-3/2's is considered:

$$\hat{H} = -J \sum_{\langle \boldsymbol{r},\boldsymbol{r}'\rangle, z} S_{\boldsymbol{r},z} \cdot S_{\boldsymbol{r}',z} + J_z \sum_{\boldsymbol{r},z} S_{\boldsymbol{r},z} \cdot S_{\boldsymbol{r},z+1} - B_z \sum_{\boldsymbol{R}} S_{\boldsymbol{R}}^z \tag{6}$$

where $J \gg J_z, B_z > 0$ are all positive parameters.

I. LARGE FIELD CASE: OUT-OF-PLANE FERROMAGNETISM

For ferromagnetism (FM) along $\hat{z}$-axis (coined $\mathrm{FM_z}$), we use the following Holstein-Primakoff representation to derive the spin wave theory:

$$S_{\boldsymbol{R}}^z = S - a_{\boldsymbol{R}}^\dagger a_{\boldsymbol{R}}, \qquad S_{\boldsymbol{R}}^+ = S_{\boldsymbol{R}}^x + iS_{\boldsymbol{R}}^y = \sqrt{2S - a_{\boldsymbol{R}}^\dagger a_{\boldsymbol{R}}} \cdot a_{\boldsymbol{R}} \tag{7}$$

In this FM_z phase, the unbroken magnetic symmetries (magnetic point group $2'/\mathrm{m}'$) are

$$\mathrm{M_x'} \equiv \mathrm{M_x} \cdot \mathrm{T}, \quad \mathrm{C_{2x}'} \equiv \mathrm{C_{2x}} \cdot \mathrm{T} \tag{8}$$

where T is the time-reversal operator. The magnon operator transforms under them as

$$a_{\boldsymbol{R}} \xrightarrow{\mathrm{M_x'}} -a_{\mathrm{M_x}\boldsymbol{R}}, \quad a_{\boldsymbol{R}} \xrightarrow{\mathrm{C_{2x}'}} -a_{\mathrm{C_{2x}}\boldsymbol{R}}, \tag{9}$$

The associated linear spin wave Hamiltonian reads

$$\hat{H}_{\mathrm{FM_z}} = (3JS + B_z - 2J_zS) \sum_{\boldsymbol{R}} a_{\boldsymbol{R}}^\dagger a_{\boldsymbol{R}} - JS \sum_{\langle \boldsymbol{r},\boldsymbol{r}'\rangle, z} a_{\boldsymbol{r},z}^\dagger a_{\boldsymbol{r}',z} + J_z S \sum_{\boldsymbol{r},z} a_{\boldsymbol{r},z}^\dagger a_{\boldsymbol{r},z+1} + h.c. \tag{10}$$

It's straightforward to obtain the two magnon branches with frequency:

$$\omega_{\boldsymbol{k},k_z,\pm} = JS(3 \mp |f_k|) + B_z - 2J_zS(1 - \cos k_z) \tag{11}$$

where $\boldsymbol{k}$ labels the in-plane 2D momentum. $f_{\boldsymbol{k}} = \sum_{j=1,2,3} e^{\mathrm{i}\boldsymbol{k}\cdot\vec{\delta}_j}$ is the structure factor of honeycomb nearest neighbors as in graphene, satisfying $f_{\boldsymbol{k}=(0,0)} = 3$. The wavefunction of the two magnon modes are given by

$$\gamma_{\boldsymbol{k},\pm} \equiv \left(a_{\boldsymbol{k},A} \pm a_{\boldsymbol{k},B}\right)/\sqrt{2} \tag{12}$$

It is straightforward to check how they transform under symmetries:

$$\gamma_{\boldsymbol{k},\pm} \xrightarrow{\mathrm{M_x'}} \mp\gamma_{-\mathrm{M_x}\boldsymbol{k},\pm}, \qquad \gamma_{\boldsymbol{k},\pm} \xrightarrow{\mathrm{C_{2x}'}} -\gamma_{-\mathrm{C_{2x}}\boldsymbol{k},\pm} \tag{13}$$

since $\mathrm{M_x}$ switches two sublattices but not $\mathrm{C_{2x}}$. For magnons at the zone center Γ, the soft $\gamma_{\Gamma,+}$ mode is odd under both symmetries and belongs to $\mathrm{B_g}$ representation of point group $2/\mathrm{m}$. On the other hand, the high frequency $\gamma_{\Gamma,-}$ mode is odd under inversion symmetry and hence not Raman active. This explains

why experiments only observe the soft $\mathrm{B_g}$ mode $\gamma_{\Gamma,+}$ mode above $B_z \geq 7$ T, but not the high frequency mode $\gamma_{\Gamma,-}$.

II. SMALL FIELD CASE: IN-PLANE ANTIFERROMAGNETISM

As suggested by the observation of SHG in bilayer CrI_3,[5] multilayer CrI_3 is likely to exhibit antiferromagnetism (AFM) between two neighboring layers. In the limit of a small magnetic field $B_z < 2$ T, we consider an in-plane moment along the $\hat{x}$-axis as reported in $CrCl_3$.[4] The Holstein-Primakoff representation writes:

$$S_{\boldsymbol{R}}^{x} = (-1)^{z} \cdot \left(S - a_{\boldsymbol{R}}^{\dagger} a_{\boldsymbol{R}}\right), \quad S_{\boldsymbol{R}}^{(-1)^{z}} \equiv S_{\boldsymbol{R}}^{x} + (-1)^{z} \mathrm{i} S_{\boldsymbol{R}}^{z} = \sqrt{2S - a_{\boldsymbol{R}}^{\dagger} a_{\boldsymbol{R}}} \cdot a_{\boldsymbol{R}} \tag{14}$$

For an even number of layers $L_z = 0 \bmod 2$, the magnetic point group is $2'/\mathrm{m}$ generated by $\mathrm{C}_{2\mathrm{x}}'$ and $\mathrm{M_x}$. The boson operator transforms as

$$a_{\boldsymbol{R}} \xrightarrow{\mathrm{M_x}} -a_{\mathrm{M_x}\boldsymbol{R}}, \quad a_{\boldsymbol{R}} \xrightarrow{\mathrm{C}_{2\mathrm{x}}'} a_{\mathrm{C}_{2\mathrm{x}}\boldsymbol{R}} \tag{15}$$

The linear spin wave Hamiltonian writes

$$\begin{aligned} \widehat{H}_{\mathrm{AFM_x}} = \sum_{\boldsymbol{R}} (3JS + 2J_z S) a_{\boldsymbol{R}}^{\dagger} a_{\boldsymbol{R}} - JS \sum_{\langle \boldsymbol{r},\boldsymbol{r}' \rangle, z} a_{\boldsymbol{r},z}^{\dagger} a_{\boldsymbol{r}',z} + J_z S \sum_{\boldsymbol{r},z} a_{\boldsymbol{r},z}^{\dagger} a_{\boldsymbol{r},z+1}^{\dagger} + h.c. \\ - \mathrm{i} B_z \sqrt{2S} \sum_{\boldsymbol{R}} (-1)^{z} \left(a_{\boldsymbol{R}}^{\dagger} - a_{\boldsymbol{R}}\right) \end{aligned} \tag{16}$$

Four branches of magnons are obtained with frequency:

$$\Omega_{\boldsymbol{k},k_z,\pm} = \sqrt{\omega_{\boldsymbol{k},\pm}\left(\omega_{\boldsymbol{k},\pm} + 4J_z S\right) + (2J_z S \sin k_z)^2} \tag{17}$$

where $\omega_{\boldsymbol{k},\pm} \equiv JS(3 \mp |f_{\boldsymbol{k}}|)$ is the FM magnon dispersion within each 2D honeycomb plane. In particular, the magnon dispersion is not affected by the out-of-plane magnetic field B_z. It would, however, be affected by an in-plane magnetic field $B_{x,y}$, which is not consistent with our experimental observations for $\mathrm{P_1}$ and $\mathrm{P_2}$

There are two soft magnon modes, $\gamma_{(0,0,0),+}$ and $\gamma_{(0,0,\pi),+}$. Their symmetry characters are summarized in Supplementary Table 2. They belong to $\mathrm{A_u}$ and $\mathrm{B_g}$ representations of group $2/\mathrm{m}$, and the $\mathrm{B_g}$ mode $\gamma_{(0,0,\pi),+}$ is the only Raman active branch of soft magnons.

If the number of layers is odd, the crystal symmetry $2/\mathrm{m}$ is fully preserved. Under symmetry operations, the bosons transform as

$$a_{\boldsymbol{R}} \xrightarrow{\mathrm{M_x}} -a_{\mathrm{M_x}\boldsymbol{R}}, \quad a_{\boldsymbol{R}} \xrightarrow{\mathrm{C_{2x}}} a_{\mathrm{C_{2x}}\boldsymbol{R}} \tag{18}$$

The associated symmetry representations of magnons are summarized in Supplementary Table 3. In this case, both soft magnons are Raman active and belong to $\mathrm{B_g}$ representation.

**Modes**	$\boldsymbol{M'_x}$	$\boldsymbol{C'_{2x}}$	**Irrep.**	**Raman active?**
$\gamma_{k_z=0,+}$	-	+	$\mathrm{A_u}$	No
$\gamma_{\pi,+}$	-	-	$\mathrm{B_g}$	Yes
$\gamma_{k_z=0,-}$	+	+	$\mathrm{A_g}$	Yes
$\gamma_{\pi,-}$	+	-	$\mathrm{B_u}$	No

Supplementary Table 2. Symmetry characters (magnetic point group $2'/\mathrm{m}$) of magnons in the $\mathrm{AFM_x}$ phase, in a thin film of CrI_3 with an even number of layers.

**Modes**	$\boldsymbol{M_x}$	$\boldsymbol{C_{2x}}$	**Irrep.**	**Raman active?**
$\gamma_{k_z=0,+}$	-	-	$\mathrm{B_g}$	Yes
$\gamma_{\pi,+}$	-	-	$\mathrm{B_g}$	Yes
$\gamma_{k_z=0,-}$	+	-	$\mathrm{B_u}$	No
$\gamma_{\pi,-}$	+	-	$\mathrm{B_u}$	No

Supplementary Table 3. Symmetry characters (magnetic point group $2/\mathrm{m}$) of magnons in the $\mathrm{AFM_x}$ phase, in a thin film of CrI_3 with an odd number of layers.

III. SMALL FIELD CASE: OUT-OF-PLANE ANTIFERROMAGNETISM

Finally, we consider out-of-plane AFM order AFM_z as a comparison to AFM_x. The calculation is straightforward and one can show its magnon spectra as

$$\Omega_{\boldsymbol{k},k_z,\pm,\eta_z=\pm 1} = \left| B_z + \eta_z \sqrt{\omega_{\boldsymbol{k},\pm}\left(\omega_{\boldsymbol{k},\pm} + 4J_z S\right) + (2J_z S \sin k_z)^2} \right| \tag{19}$$

Clearly, all magnon frequencies shift with the applied magnetic field B_z, which is not consistent with our data for $\mathrm{P_1}$ and $\mathrm{P_2}$.

For comparison, we also list the representations of magnon modes at zone center Γ, for the case of even (Supplementary Table 4) vs. odd (Supplementary Table 5) layers in the thin film. They are the same as in the AFM_x phase, although their magnetic point groups are different from the AFM_x phase.

Modes	M_x'	C_{2x}	Irrep.	Raman active?
$\gamma_{k_z=0,+}$	-	+	A_u	No
$\gamma_{\pi,+}$	-	-	B_g	Yes
$\gamma_{k_z=0,-}$	+	+	A_g	Yes
$\gamma_{\pi,-}$	+	-	B_u	No

Supplementary Table 4. Symmetry characters (magnetic point group $2'/m'$) of magnons in the AFM_z phase, in a thin film of CrI_3 with an even number of layers.

Modes	M_x'	C_{2x}'	Irrep.	Raman active?
$\gamma_{k_z=0,+}$	-	-	B_g	Yes
$\gamma_{\pi,+}$	-	-	B_g	Yes
$\gamma_{k_z=0,-}$	+	-	B_u	No
$\gamma_{\pi,-}$	+	-	B_u	No

Supplementary Table 5. Symmetry characters (magnetic point group $2'/m'$) of magnons in the AFM_z phase, in a thin film of CrI_3 with an odd number of layers.

In summary, if $P_{1,2}$ were due to the bound state of a magnon and a phonon where the spins were aligned in-plane, they would not be expected to shift in frequency under the application of a magnetic field perpendicular to the ab plane, which follows the observed behavior of $P_{1,2}$. They would, however, be expected to display a shift in frequency when the magnetic field was applied in the ab plane, but this is not consistent with what was observed in Supplementary Figure 10. Thus, we have ruled out that $P_{1,2}$ are due to the bound state of a magnon and a phonon.

Supplementary Note 3: P_1 and P_2 as Zone-Folded Phonons

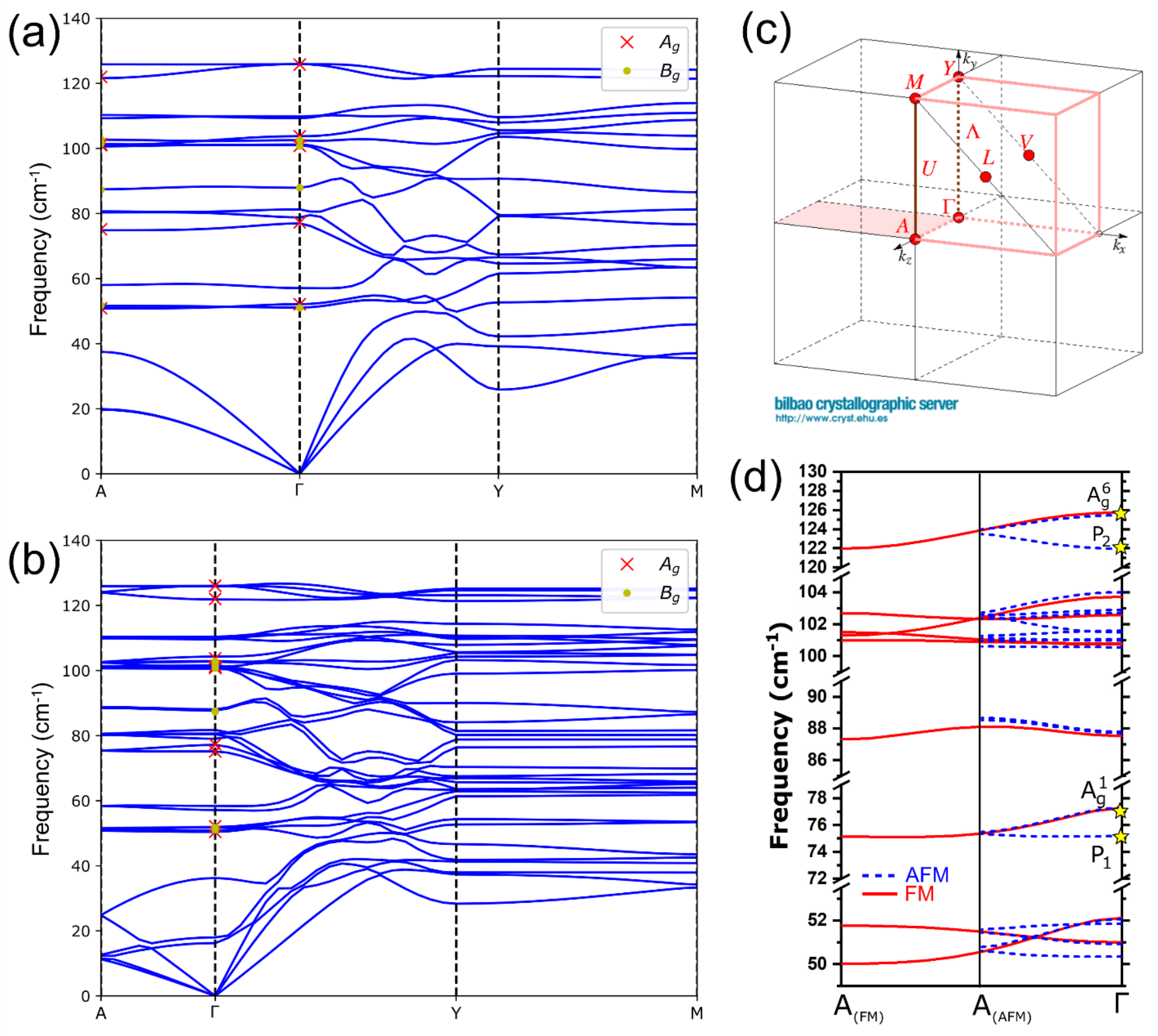

Supplementary Figure 12: Calculated phonon dispersion of (a) ferromagnetic (FM) and (b) antiferromagnetic (AFM) stacking for bulk CrI_3 in the monoclinic crystal structure. The relevant Brillouin zone from Bilbao crystallographic server[6-8] is shown in (c). In the AFM case, the unit cell doubles along the c-direction in real space, such that the A-point folds into Γ and the number of modes is doubled compared to the FM case. The symmetry of the Raman-active modes is marked on the phonon dispersion as A_g or B_g at the Γ point. It should be noted that a constant number of points was used between the high symmetry k-points for both the FM and AFM cases even though the size of the Brillouin zone in the Γ – A direction is half in the AFM vs. FM cases. Thus, it is not possible to visually compare the derivatives of the acoustic modes at Γ in order to get the speed of sound. (d) Comparing the calculated phonon dispersion showing the Raman-active modes between 45 cm^{-1} and 130 cm^{-1} in the FM (red, solid) and AFM (blue, dashed) stacking configurations. The Γ – A distance in the AFM case is half that of the FM case.

Ferromagnetic (FM)		Antiferromagnetic (AFM)		
**Frequency**	**Q-Point**	**Frequency**	**Q-Point**	**Character**
50.02	A	50.35	Γ	oo-phase
51.00	Γ	50.93	Γ	in-phase
51.77	A	51.85	Γ	oo-phase
52.12	Γ	52.07	Γ	in-phase
75.15	A	75.17	Γ	oo-phase
77.28	Γ	77.32	Γ	in-phase
87.31	A	87.70	Γ	oo-phase
87.53	Γ	87.80	Γ	in-phase
100.80	Γ	100.57	Γ	in-phase
100.73	Γ	100.98	Γ	in-phase
100.98	A	101.07	Γ	oo-phase
101.32	A	101.49	Γ	oo-phase
101.51	A	101.60	Γ	oo-phase
102.59	Γ	102.73	Γ	mix-phase
102.70	A	102.90	Γ	mix-phase
103.74	Γ	104.02	Γ	in-phase
121.98	A	121.96	Γ	oo-phase
125.77	Γ	125.47	Γ	in-phase
202.06	Γ	202.55	Γ	in-phase
201.87	A	202.67	Γ	oo-phase
225.54	A	226.31	Γ	oo-phase
226.36	Γ	226.70	Γ	in-phase
226.79	A	227.14	Γ	oo-phase
226.87	Γ	227.47	Γ	in-phase

Supplementary Table 6: DFT-calculated phonon frequency and Q-points for the Raman-active phonons in monoclinically-stacked, bulk CrI_3 for FM and AFM interlayer exchange coupling. For FM exchange, we list the phonon frequencies and symmetries at the A-point in the Brillouin zone as well, which is along the *c*-direction in real space. In the AFM state, the unit cell doubles and the A-point is folded into Γ and thus can become Raman-active. In general, the splitting between the mode originally at Γ and the mode zone-folded from the A-point are very small and most likely not resolvable. However, for the pairs of modes highlighted in red, the splitting is significant, with the mode from A at a lower frequency than the one from Γ.

Frequency (cm^{-1})	Q-Point in FM	Symmetry	Vibration
75.2	A	B_u	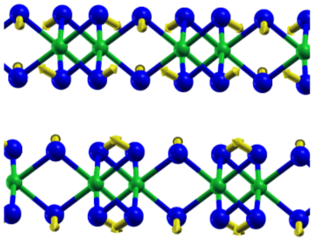
77.3	Γ	A_g	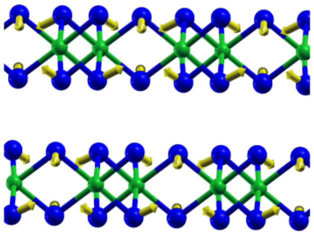
122.0	A	B_u	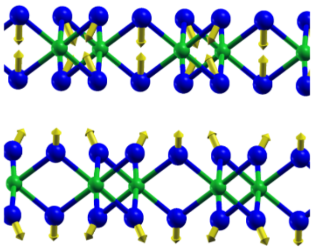
125.8	Γ	A_g	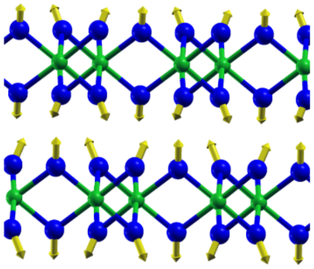

Supplementary Figure 13: DFT-calculated phonon vibrations for A_g^1 and A_g^6 as well as the zone-folded phonons at lower frequency. Eigenvectors are calculated for a bulk system but are pictured as a bilayer system. Looking at the vibrations for a bilayer system, the two zone-folded vibrations would have B_u symmetry with the inversion center in-between the two layers. When magnetic ordering is considered, however, where the two layers have AFM stacking, there is no longer inversion symmetry and the modes will be of B symmetry, and thus can be Raman active.

Supplementary References:

1 Wang, Z. *et al.* Very large tunneling magnetoresistance in layered magnetic semiconductor CrI_3. *Nat. Commun.* **9**, 2516 (2018).

2 Pradhan, S., Patel, N. D. & Trivedi, N. Two-magnon bound states in the Kitaev model in a [111] field. *Phys. Rev. B* **101**, 180401(R) (2020).

3 Lee, I. *et al.* Fundamental spin interactions underlying the magnetic anisotropy in the Kitaev ferromagnet CrI_3. *Phys. Rev. Lett*. **124**, 017201 (2020)

4 Klein, D. R. *et al.* Enhancement of interlayer exchange in an ultrathin two-dimensional magnet. *Nat. Phys.* 1255-1260 (2019).

5 Sun, Z. *et al.* Giant nonreciprocal second-harmonic generation from antiferromagnetic bilayer CrI_3. *Nature* **572**, 497-501 (2019).

6 Aroyo, M. I. *et al.* Crystallography online: Bilbao Crystallographic Server. *Bulgarian Chemical Communications* **43**, 183-197 (2011).

7 Aroyo, M. I. *et al.* Bilbao crystallographic server: I. Databases and crystallographic computing programs. *Zeitschrift Fur Kristallographie* **221**, 15-27 (2006).

8 Aroyo, M. I., Kirov, A., Capillas, C., Perez-Mato, J. M. & Wondratschek, H. Bilbao crystallographic server. II. Representations of crystallographic point groups and space groups. *Acta Crystallographica a-Foundation and Advances* **62**, 115-128 (2006).
